# LOOP, a cross-sectional, observational study investigating the clinical specialty setting as a determinant of disease management in psoriatic arthritis: Subgroup analysis results from Japan

**DOI:** 10.1371/journal.pone.0245954

**Published:** 2021-01-27

**Authors:** Masato Okada, Sarina Kurimoto, Fabiana Ganz, Wolf-Henning Boehncke

**Affiliations:** 1 Division of Allergy and Rheumatology, Immuno-Rheumatology Center, St. Luke’s International Hospital, Tokyo, Japan; 2 Immunology, Medical, AbbVie GK, Tokyo, Japan; 3 Research & Development, Global Development Project, AbbVie AG, Baar, Switzerland; 4 Division of Dermatology and Venereology, Geneva University Hospitals, Geneva, Switzerland; Universita Campus Bio-Medico di Roma, ITALY

## Abstract

**Background:**

Psoriatic arthritis (PsA) is a progressive erosive joint disorder that causes functional impairment; therefore, early diagnosis and management are essential. This study evaluated the association between clinical specialty and the time to management in patients with PsA in Japan.

**Methods:**

This was a subgroup analysis of a cross-sectional, multicenter, observational study that was conducted in 17 countries outside the United States, including 17 sites at 8 institutions in Japan, from June 2016 to October 2017. Data from consecutive patients (age ≥18 years) with a suspected or established diagnosis of PsA on a routine visit to a participating rheumatology/orthopedic or dermatology clinic in Japan were analyzed. The primary endpoints were time from onset of inflammatory musculoskeletal symptoms to PsA diagnosis, PsA diagnosis to first conventional synthetic disease-modifying antirheumatic drug (csDMARD), PsA diagnosis to first biologic DMARD (bDMARD), and first csDMARD to first bDMARD.

**Results:**

Of 109 patients with a confirmed diagnosis of PsA, 39.4% (n = 43) and 60.6% (n = 66) were recruited by rheumatologists/orthopedists and dermatologists, respectively. Most patients were prescribed tumor necrosis factor inhibitors (58.7%) or methotrexate (56.0%). The mean duration from symptom onset to PsA diagnosis was significantly longer (p = 0.044) for patients treated by rheumatologists/orthopedists (70.6 months) than those treated by dermatologists (30.1 months). In the rheumatology/orthopedic and dermatology settings, the mean time from PsA diagnosis to first csDMARD administration was −0.9 and −2.9 months, and from PsA diagnosis to first bDMARD 21.4 and 14.9 months, respectively. The mean duration from administration of first csDMARD to first bDMARD was comparable in the rheumatology/orthopedic (31.8 months) and dermatology (31.5 months) settings.

**Conclusions:**

Treatment approach was slightly different between rheumatology/orthopedic and dermatology setting in clinical practice in Japan, suggesting that an integrated dermo-rheumatologic approach can optimize the management of patients with PsA.

## Introduction

Psoriasis (PsO) is a prevalent skin condition that often affects the joints, leading to psoriatic arthritis (PsA) [1]. The global prevalence of PsA among patients with PsO is estimated to be between 6% and 42% [2]. Previously, the prevalence of PsA in patients with PsO was reported as <1% in the Japanese population [2]. However, recent studies suggest a prevalence of approximately 15% [2,3], clearly indicating that PsA is common among patients with PsO in Japan and that underdiagnosis could be one of the reasons for the previously reported low prevalence.

PsA is a progressive erosive joint disorder that causes functional impairment in the majority of patients; therefore, early diagnosis and management are essential to prevent disability and improve long-term outcomes [4]. Notably, since PsA symptoms tend to appear several years after the onset of symptoms of cutaneous PsO, patients will often present to a dermatologist for treatment of PsO. Therefore, dermatologists play a pivotal role in screening for signs of PsA, early diagnosis, treatment initiation, and timely referral of patients to a rheumatologist [5,6]. According to a study in the United Kingdom, nearly 50% of referrals from a dermatology to a rheumatology clinic involved patients with PsO and suspected PsA [7]. However, studies conducted in dermatology clinics across Europe and North America reported the prevalence of undiagnosed PsA in patients with PsO to be as high as 41%, highlighting the challenge of diagnosing PsA in this setting [8,9]. Thus, the timely diagnosis and optimal management of PsA potentially require a multidisciplinary approach involving both dermatologists and rheumatologists [10]. Evidence from previous studies has shown that a successful collaboration between dermatologists and rheumatologists leads to improved management of patients with PsA, resulting in clinical remission and a significant improvement in a patient’s quality of life [11–13].

To gain further insights into factors influencing the management of PsA, the LOOP study [14] investigated the association between clinical specialty and time to management in patients with a confirmed diagnosis of PsA in several countries, including Japan. Among 1273 patients with confirmed PsA in the LOOP study, when comparing patients who were seen by a rheumatologist or a dermatologist, the median time from onset of inflammatory musculoskeletal symptoms to PsA diagnosis was not significantly different (6.0 *vs*. 3.9 months, respectively), and the median time from diagnosis to first conventional synthetic disease-modifying antirheumatic drug (csDMARD) treatment was significantly shorter (0 *vs*. 2.0 months; p < 0.001, respectively). In addition, patients assessed by a dermatologist presented with higher levels disease activity [14]. These results demonstrated the importance of a multidisciplinary approach towards disease management in patients with PsA, which has also been discussed in previous studies [11–13].

Similar to other countries, in Japan, PsA is diagnosed or treated in either a dermatology or a rheumatology setting [15]. However, unlike in other countries, orthopedists and rheumatologists can treat patients with PsA with or without surgical intervention. Certified rheumatologists include those certified by the Japan College of Rheumatology and/or those certified by the Japanese Orthopedic Association. In Japan, in addition to the medical treatment provided by a rheumatologist, an orthopedic rheumatologist provides surgical treatment. A subgroup analysis of the LOOP study was performed to assess differences between rheumatology/orthopedic and dermatology settings in terms of PsA diagnosis, management approach, and characteristics of patients with PsA in clinical practice in Japan

## Methods

### Study design and patient population

In Japan, the LOOP study was conducted at 17 sites (departments) at 8 institutions from June 2016 to October 2017. The number of patients was not prespecified for this subgroup analysis. Collaboration categories were defined as “newly formed for this study,” “established relationship,” and “no relationship.” Among participating sites, 15 were at university hospitals and had established relationships, and 2 were at private hospitals; the relationship between these 2 hospitals was newly formed for this study. The study duration was 16 to 18 months.

For the management of patients with PsA, regular collaboration between different specialties was advised per established clinical recommendations [16–21]. To ensure accurate and standardized assessments of joint and skin disease activity scores, patients recruited by a rheumatologist/orthopedist were advised to seek consultation with a dermatologist and vice versa. Data were obtained from data-recording forms collected centrally at GKM: Gesellschaft für Therapieforschung mbH, München, Germany. To maintain patient confidentiality, demographic data (except age) that could identify individuals were not collected.

Consecutive adult patients (age ≥18 years) with a suspected or established diagnosis of PsA on a routine clinical visit to a participating rheumatology/orthopedic/rheumatology or dermatology site and who signed a patient authorization/informed consent form for the use and disclosure of their anonymized personal health information were eligible to participate in the study. All patients were required to read and understand the patient questionnaires in Japanese. English and Japanese versions of the patient questionnaires that were coded with a unique patient number were provided to each site in paper format. All enrolled patients were assessed by both a rheumatologist/orthopedist and a dermatologist. The diagnosis of PsA was confirmed using the ClASsification criteria for Psoriatic ARthritis (CASPAR) [22].

This study was conducted in compliance with Japanese laws and regulations, including the Personal Information Protection Act and ethical guidelines on medical research targeting humans. The responsible Ethics Committee and Health Institutions were notified as required by Japanese laws and regulations. The study was approved by the ethics committees/institutional review board(s) of The University of Tokyo for Epidemiologic and Observational Research, Tohoku University Graduate School of Medicine, Osaka University Hospital, Matsuyama Red Cross Hospital, Hokkaido University Hospital, Osaka City University Graduate School of Medicine, Toho University Omori Medical Center, Fukuoka University. This study was registered in the UMIN clinical trials registry of Japan (UMIN000023510).

### Study endpoints and assessments

The primary endpoints were time from onset of inflammatory musculoskeletal symptoms to PsA diagnosis, time from PsA diagnosis to first csDMARD, time from PsA diagnosis to first biologic DMARD (bDMARD), and time from first csDMARD to first bDMARD.

Secondary endpoints included assessment of joint disease activity (disease activity in PsA, minimal disease activity, 28-joint disease activity score, tender joint count 68, and swollen joint count [SJC] 66); skin disease activity (body surface area of psoriasis [BSA] %, physician global assessment [PhGA] score, and number of tender entheseal points, digits with dactylitis, and nails with psoriatic changes); and disease burden (Health Assessment Questionnaire-Disability Index [HAQ-DI], Short Form-12 version 2 [SF-12], Work Productivity and Activity Impairment in psoriatic arthritis [WPAI-PsA], and Dermatology Life Quality Index [DLQI]).

Data, including demographic information, physical examination data, PsA symptoms and diagnosis according to CASPAR, medical history, comorbidities, and treatments for PsA, were documented at all recruiting sites. The following data were also collected at the recruiting sites: patients’ age, clinical specialty setting (rheumatology/orthopedic or dermatology) of the recruiting site, if a PsA diagnosis was suggested, and whether a suspected or established diagnosis was made for patients with a suggested PsA diagnosis. Rheumatological assessments included collection of data on confirmation of PsA diagnosis according to CASPAR, musculoskeletal assessment, BSA (%), PhGA score, and treatment change for PsA. Dermatological assessments included collection of data on PsO (BSA [%] and number of psoriatic nails), confirmation of PsO and PsA diagnosis, musculoskeletal signs, and PhGA score.

Information on disease burden was collected using the Japanese versions of patient-reported outcome questionnaires that were administered in paper format. The 12-item Short Form-12 version 2^®^ Health Survey Standard, Japan (in Japanese), including the physical and mental component summaries, was used to assess patients’ sense of well-being, feelings, and ability to perform daily activities over the preceding 4 weeks. Using a recall period of 1 week, the 15-item HAQ-DI was used to assess patients’ physical function and health-related quality of life, the 6-item WPAI-PsA was used to assess the impact of PsA on patients’ ability to work and perform activities of daily living, and the 10-item DLQI questionnaire was used to measure the impact of skin disease on patients’ lives.

### Statistical analysis

Clinical specialty was defined as follows: “dermatology” if a specialist was either a dermatologist or a specialist other than a dermatologist/orthopedist/rheumatologist with certified training in dermatology, and “rheumatology/orthopedic” if a specialist was either a rheumatologist/orthopedist or a specialist other than an rheumatologist/orthopedist/dermatologist with certified training in rheumatology.

Patients who fulfilled the inclusion criteria and had a confirmed diagnosis of PsA were included in this analysis.

Quantitative data were summarized as N, missing N, mean, standard deviation (SD), minimum (0%), median (50%), and maximum (100%). Because of the observational nature of this study, no adjustment for multiplicity was made and no safety analyses were performed. A simple linear regression analysis by clinical specialty, adjusted for age and sex, was conducted to calculate mean differences for all primary and secondary endpoints. Inferential statistical analyses were conducted at a nominal 2-sided significance level of 0.05. In addition, differences in baseline patient characteristics, clinical history, and PsA treatment by enrolling clinical specialty were further investigated. Wilcoxon rank-sum test was used for the comparison of continuous variables, clinical history, and PsA treatment. Fisher's exact test was used for the comparison of categorical variables. Statistical analyses were performed using SAS^®^ package (version 9.2 or higher) or software package R if functionality was not available in SAS.

## Results

### Patient disposition

Overall, 111 Japanese patients with a suspected or established diagnosis of PsA were enrolled. Of these, 109 patients (98.2%) were included in this subgroup analysis and 2 (1.8%) were excluded due to absence of a confirmed PsA diagnosis. More than 50% of patients were recruited by dermatologists (60.6%, n = 66) compared with 39.4% (n = 43) who were am recruited by rheumatologists/orthopedists. The proportion of patients with an established diagnosis of PsA was comparable between those recruited by rheumatologists/orthopedists and those recruited by dermatologists (97.7% and 97.0%, respectively).

### Patient characteristics, clinical history, and PsA treatment by enrolling clinical specialty

Demographics and disease characteristics of patients with PsA by enrolling clinical specialty are shown in Table 1. The percentages of patients with a clinical history of PsO (88.4% *vs*. 98.5%, p = 0.034) and dactylitis (60.5% *vs*. 81.8%, p = 0.016) were lower in the rheumatology/orthopedic setting than in the dermatology setting. Also, patients had a numerically lower mean (SD) body mass index (23.5 [3.6] kg/m^2^ vs 25.3 [4.4] kg/m^2^, p = 0.055) and the percentage of obese patients (4.7% vs 16.7%, p = 0.073) was numerically lower in the rheumatology/orthopedic setting than in the dermatology setting.

**Table 1:** Demographics, disease characteristics, and clinical history of patients with psoriatic arthritis by recruiting clinical specialty in Japan.

Characteristic	Overall (N = 109)	Rheumatology/Orthopedic (n = 43)	Dermatology (n = 66)	p-value
**Age, years, mean (SD)**	55.9 (13.5)	58.3 (13.9)	54.4 (13.1)	0.147
**Sex, male, n (%)**	62 (56.9)	26 (60.5)	36 (54.5)	0.560
**Weight, kg, mean (SD)**	66.2 (14.9)	63.5 (12.1)	67.9 (16.3)	0.191
**BMI, kg/m**^**2**^**, mean (SD)**	24.6 (4.1)	23.5 (3.6)	25.3 (4.4)	0.055
**Time from PsA diagnosis to recruiting site visit, months**				
**Mean (SD)**	54.3 (89.8)	52.2 (88.4)	55.6 (91.3)	0.505
**Median**	22.8	20.1	24.2	–
**Current** ^**a**^ **skin symptoms, n (%)**	107 (98.2)	41 (95.3)	66 (100.0)	0.153
**Current** ^**a**^ **enthesitis, n (%)**	45 (41.3)	15 (34.9)	30 (45.5)	0.322
**Current** ^**a**^ **dactylitis, n (%)**	93 (85.3)	35 (81.4)	58 (87.9)	0.411
**Current** ^**a**^ **swollen joints, n (%)**	101 (92.7)	38 (88.4)	63 (95.5)	0.260
**Family history of psoriasis, n (%)**	9 (8.3)	5 (11.6)	4 (6.1)	0.313
**Any relevant personal medical history, n (%)**	106 (97.2)	41 (95.3)	65 (98.5)	0.561
**Psoriasis**	103 (94.5)	38 (88.4)	65 (98.5)	0.034
**Dactylitis**	80 (73.4)	26 (60.5)	54 (81.8)	0.016
**Enthesitis**	51 (46.8)	16 (37.2)	35 (53.0)	0.120
**Axial disease**	31 (28.4)	8 (18.6)	23 (34.8)	0.083
**Uveitis**	7 (6.4)	3 (7.0)	4 (6.1)	1.000
**Inflammatory bowel disease**	3 (2.8)	1 (2.3)	2 (3.0)	1.000
**Any relevant current comorbidity, n (%)**	74 (67.9)	25 (58.1)	49 (74.2)	0.095
**Hypertension**	51 (46.8)	17 (39.5)	34 (51.5)	0.244
**Lipid disorder**	36 (33.0)	12 (27.9)	24 (36.4)	0.409
**Obesity**	13 (11.9)	2 (4.7)	11 (16.7)	0.073
**Depression and/or anxiety**	8 (7.3)	3 (7.0)	5 (7.6)	1.000
**Type II diabetes**	22 (20.2)	7 (16.3)	15 (22.7)	0.472
**Cardiovascular disease**	17 (15.6)	9 (20.9)	8 (12.1)	0.281
**Osteoporosis**	11 (10.1)	6 (14.0)	5 (7.6)	0.337

BMI, body mass index; PsA, psoriatic arthritis; SD, standard deviation.

^a^ Anytime during the disease course.

Overall, 95.4% of patients received treatment for PsA; 97.7% were treated by rheumatologists/orthopedists and 93.9% by dermatologists (Table 2). Most patients were prescribed tumor necrosis factor inhibitors (TNFi; 58.7%) and/or methotrexate (56.0%). Prescribing behavior for TNFi was comparable (55.8% *vs*. 60.6%, p = 0.692) between rheumatologists/orthopedists and dermatologists, while a lower percentage of patients were prescribed anti–interleukin (IL)-12/23 or other bDMARDs (7.0% *vs*. 25.8%, p = 0.021) and cyclosporin (4.7% *vs*. 22.7%, p = 0.014), respectively. The most frequently prescribed first csDMARD was methotrexate (70.8%), with a higher frequency of prescriptions by rheumatologists/orthopedists (83.9% vs 61.0%, p = 0.040).

**Table 2:** Psoriatic arthritis treatment by enrolling clinical specialty in Japan.

Treatment, n (%)	Overall (N = 109)	Rheumatology/Orthopedic (n = 43)	Dermatology (n = 66)	p-value
**Any treatment**	104 (95.4)	42 (97.7)	62 (93.9)	0.646
**All treatments (in >10% of patients)**				
** Methotrexate**	61 (56.0)	27 (62.8)	34 (51.5)	0.324
** TNFi**	64 (58.7)	24 (55.8)	40 (60.6)	0.692
** Sulfasalazine**	24 (22.0)	13 (30.2)	11 (16.7)	0.104
** Systemic steroids**	15 (13.8)	7 (16.3)	8 (12.1)	0.578
** Leflunomide**	1 (0.9)	1 (2.3)	0 (0.0)	0.394
** Anti**–**IL-12/23 or other bDMARDs**	20 (18.3)	3 (7.0)	17 (25.8)	0.021
** Cyclosporin**	17 (15.6)	2 (4.7)	15 (22.7)	0.014
**Number of patients on first csDMARD**	72	31	41	0.308
**First csDMARD** ^**a**^ **(in >10% of patients)**				
** Methotrexate**	51 (70.8)	26 (83.9)	25 (61.0)	0.040
** Sulfasalazine**	8 (11.1)	4 (12.9)	4 (9.8)	0.719
**Currently still on first csDMARD**	47 (43.1)	28 (65.1)	19 (28.8)	<0.001
** Methotrexate** ^**b**^	37 (78.7)	24 (85.7)	13 (68.4)	0.276
** Sulfasalazine** ^**b**^	5 (10.6)	3 (10.7)	2 (10.5)	1.000
**Number of patients on first bDMARD**	71	27	44	0.687
**First bDMARD** ^**c**^ **(in >10% of patients)**				
** TNFi**	63 (88.7)	24 (88.9)	39 (88.6)	1.000
** Anti**–**IL-12/23 or other bDMARDs**	8 (11.3)	3 (11.1)	5 (11.4)	1.000
**Currently still on first bDMARD**	49 (45.0)	21 (48.8)	28 (42.4)	0.558
** TNFi** ^**d**^	42 (85.7)	18 (85.7)	24 (85.7)	1.000
** Anti**–**IL-12/23 or other bDMARDs** ^**d**^	7 (14.3)	3 (14.3)	4 (14.3)	1.000

Anti–IL-12/23, anti-interleukin 12 or anti-interleukin 23; bDMARD, biologic disease-modifying antirheumatic drug; csDMARD, conventional synthetic disease-modifying antirheumatic drug; TNFi, tumor necrosis factor inhibitor.

^a^ Subset of total patients on first csDMARD.

^b^ Subset of total patients currently still on first csDMARD.

^c^ Subset of total patients on first bDMARD.

^d^ Subset of total patients currently still on first bDMARD.

### Time to disease management by enrolling clinical specialty

**Fig 1:**
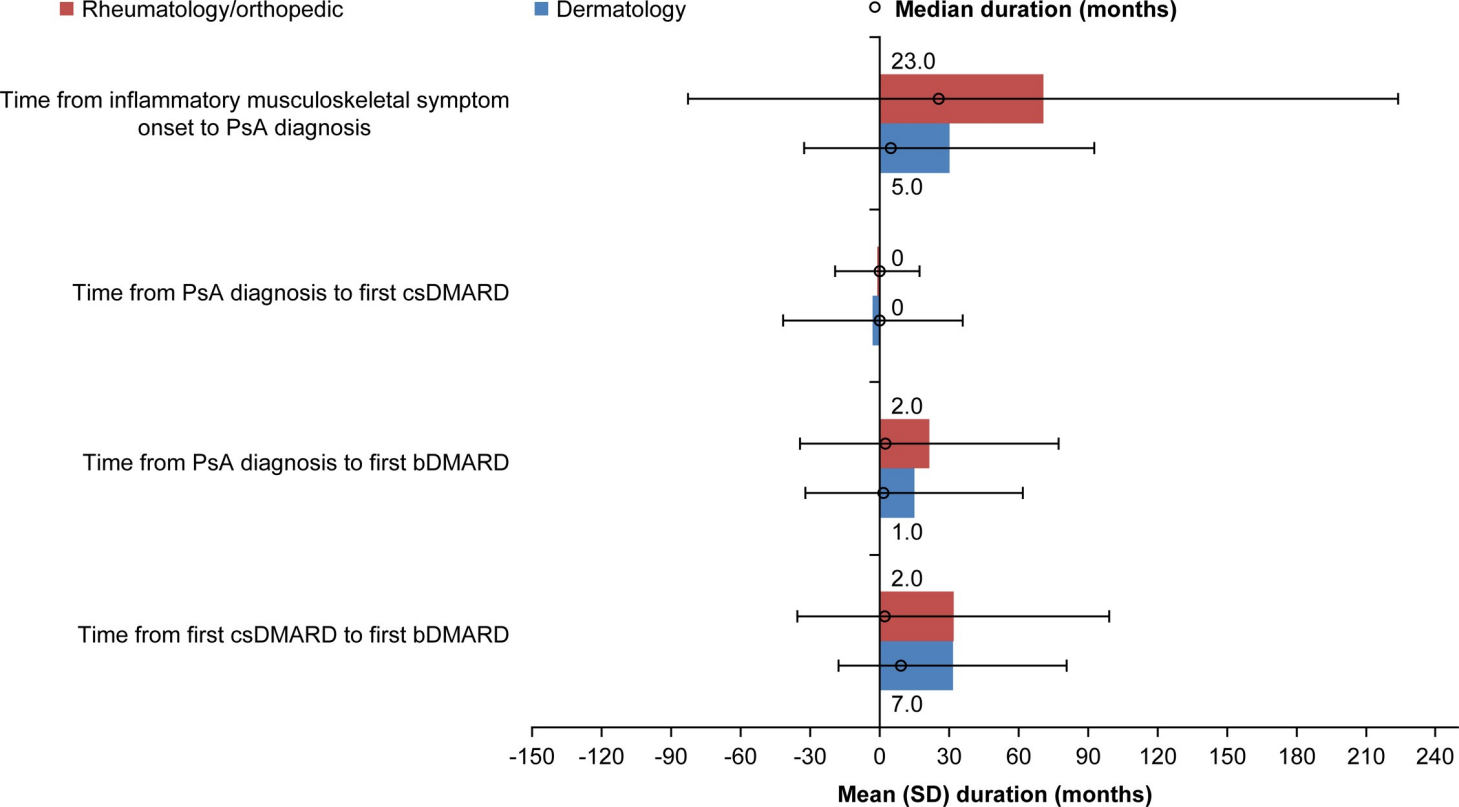
Time to disease management by enrolling clinical specialty in Japan. bDMARD, biologic disease-modifying antirheumatic drug; csDMARD, conventional synthetic disease-modifying antirheumatic drug; PsA, psoriatic arthritis; SD, standard deviation.

In Japan, the mean (SD) duration from onset of inflammatory musculoskeletal symptoms to PsA diagnosis was significantly longer (Fig 1) (p = 0.044) if the patient was diagnosed by a rheumatologist/orthopedist (70.6 [153.2] months; median, 23.0 months) than a dermatologist (30.1 [62.6] months; median, 5.0 months) (Table 3). Patients in Japan received their first csDMARD in a mean (SD) time of −0.9 (18.2) and −2.9 (38.8) months from PsA diagnosis from rheumatologists/orthopedists and dermatologists, respectively. The mean (SD) time from PsA diagnosis to the first bDMARD was 21.4 (55.8) and 14.9 (46.8) months, respectively, and the median time was 2.0 months and 1.0 month, respectively. Although the mean (SD) time from first csDMARD to first bDMARD was comparable between rheumatologists/orthopedists (31.8 [67.4] months) and dermatologists (31.5 [49.2] months), the median duration was 2.0 and 7.0 months, respectively.

**Table 3:** Timing of disease management by enrolling clinical specialty in Japan.

Duration, months	Rheumatology/Orthopedic	Dermatology	
**Time from onset of inflammatory musculoskeletal symptoms to PsA diagnosis, months**	40	63	n = 103
** Mean (SD)**	70.6 (153.2)	30.1 (62.6)	p value **=** 0.0440
** Median (range)** ^a^	23.0 (−489.0 to 535.0)	5.0 (−48.0 to 291.0)	–
**Time from PsA diagnosis to first csDMARD, months**	30	40	n = 70
** Mean (SD)**	−0.9 ^b^ (18.2)	−2.9 ^a^ (38.8)	p value **=** 0.8055
** Median (range)** ^a^	0 (−75.0 to 33.0)	0 (–135.0 to 108.0)	–
**Time from PsA diagnosis to first bDMARD, months**	27	44	n = 71
** Mean (SD)**	21.4 (55.8)	14.9 (46.8)	p value **=** 0.6358
** Median (range)** ^a^	2.0 (−26.0 to 232.1)	1.0 (−77.0 to 189.0)	–
**Time from first csDMARD to first bDMARD, months**	17	25	n = 42
** Mean (SD)**	31.8 (67.4)	31.5 (49.2)	p value **=** 0.8731
** Median (range)** ^a^	2.0 (−24.0 to 232.1)	7.0 (−12.0 to 171.0)	–

bDMARD, biologic disease-modifying antirheumatic drug; csDMARD, conventional synthetic disease-modifying antirheumatic drug; PsA, psoriatic arthritis; SD, standard deviation.

^a^ Linear regression analysis was not conducted for median (range) values.

^b^ Negative values indicate that the first csDMARD was started before PsA diagnosis.

### Assessment of disease activity by enrolling clinical specialty in Japan

Current disease activity was generally comparable in patients treated by rheumatologists/orthopedists and dermatologists in Japan, although SJC 66 (2.6 vs 1.4, respectively, p = 0.043) and the number of nails with psoriatic changes (4.1 vs 7.9, respectively, p = 0.0038) were significantly different between the two specialties. There was no significant difference in the disease burden score reported by patients managed by either clinical specialties (Table 4).

**Table 4:** Current disease activity and burden by enrolling clinical specialty in Japan.

Disease measure ^a^	Rheumatology/Orthopedic (n = 43)	Dermatology (n = 66)	
**TJC68**	n = 43	n = 65	n = 108
	4.3 (7.8)	3.2 (5.7)	p-value = 0.3571
**SJC66**	n = 43	n = 64	n = 107
	2.6 (4.5)	1.4 (2.1)	p-value = 0.043
**Tender entheseal points**	n = 43	n = 65	n = 108
	0.8 (1.6)	0.5 (1.6)	p-value = 0.2594
**Dactylitis count**	n = 43	n = 65	n = 108
	1.0 (2.7)	1.2 (4.3)	p-value = 0.9726
**PhGA**	n = 43	n = 66	n = 109
	2.7 (3.1)	3.2 (2.6)	p-value = 0.3634
**BSA (%)**	n = 43	n = 66	n = 109
	4.2 (5.7)	5.4 (10.3)	p-value = 0.4952
**Number of nails with psoriatic changes**	n = 43	n = 66	n = 109
	4.1 (5.7)	7.9 (7.8)	p-value = 0.0038
**DAPSA**	n = 41	n = 55	n = 96
	14.1 (18.8)	10.7 (11.9)	p-value = 0.2267
**DAS28**	n = 26	n = 52	n = 78
	2.9 (1.3)	2.3 (1.1)	p-value = 0.0508
**MDA present, n (%)**	n = 41	n = 58	n = 99
	24 (58.5)	31 (53.4)	p-value = 0.4695
**HAQ-DI**	n = 43	n = 62	n = 105
	0.6 (0.7)	0.5 (0.5)	p-value = 0.5326
**SF12v2, n**	n = 43	n = 63	n = 106
** PCS**	47.1 (8.6)	46.1 (9.0)	p-value = 0.6088
** MCS**	49.1 (12)	49.9 (9.1)	p-value = 0.5616
**WPAI-PsA, TWPI (%)**	n = 19	n = 30	n = 49
	23.8 (29.3)	19.4 (24.6)	p-value = 0.3391
**WPAI-PsA, TAI (%)**	n = 43	n = 64	n = 107
	27.0 (31.3)	27.7 (27.5)	p-value = 0.8318
**DLQI**	n = 42	n = 65	n = 107
	4.6 (5.5)	3.8 (3.4)	p-value = 0.2866

BSA, body surface area of psoriasis; DAPSA, disease activity in psoriatic arthritis; DAS28, 28-joint disease activity score; DLQI, Dermatology Life Quality Index; HAQ-DI, Health Assessment Questionnaire-Disability Index; MCS, mental component summary; MDA, minimal disease activity; PCS, physical component summary; PhGA, physician global assessment; SD, standard deviation; SF12v2, Short Form-12 version 2; SJC, swollen joint count; TAI, total activity impairment; TJC, tender joint count; TWPI, total work productivity impairment; WPAI-PsA, Work Productivity and Activity Impairment in psoriatic arthritis.

^a^ Data are mean (SD) unless otherwise indicated.

## Discussion

To the best of our knowledge, this is the first study in Japanese patients to investigate the association between clinical specialty and the time to and nature of disease management.

It was observed that PsA diagnosis from onset of symptoms was delayed in Japan in the rheumatology/orthopedic setting (median time: 23 months) compared with that in the dermatology setting (median time: 5 months). There could be several reasons for such a delay. Firstly, patients with PsA tend to visit rheumatologists only at an advanced stage, namely after the onset of joint pain, which represents a prolonged time from affliction to consultation with a rheumatologist. Secondly, the Japanese government health insurance system reimburses medical expenses for management of joint pain by osteopaths or physiotherapists. Although alternative medical practitioners are not trained to diagnose PsA and the scientific validity of alternative therapy is not proven, many patients prefer to consult alternative specialties for management of joint pain first [23]. This may result in a delayed referral to a rheumatologist/orthopedist, which could lead to a delayed diagnosis/treatment of PsA in Japan. Evidence has shown that a 6-month delay in referral can worsen functional outcomes [24]; therefore, early referral of patients to an orthopedist/rheumatologist needs to be facilitated.

Rheumatologists provided any treatment more frequently than dermatologists in the overall LOOP population (97.2% *vs*. 81.0%) [14], however, the difference between treating specialists was small and not clinically relevant in Japan (97.7% *vs*. 93.9%).

In Japan, rheumatologists/orthopedists prescribed methotrexate most frequently, possibly as a result of prescribing behaviors when treating patients with rheumatoid arthritis [25]. Methotrexate use was not as frequent in the dermatology setting as in the rheumatology/orthopedic setting although it was preferred over that of bDMARDs among dermatologists, likely because it was recently (2018) approved in Japan for PsO and PsA by a public domain application [26]. The prescription rate of cyclosporin was <5% for rheumatologists compared with approximately 23% for dermatologists, possibly due to cyclosporin being commonly used by dermatologists in the treatment of moderate to severe PsO [27]. On the other hand, sulfasalazine is not very effective against PsO, and thus, may not be preferentially selected as a treatment by dermatologists. In addition, the negligible use of leflunomide is likely due to it being approved only for the treatment of rheumatoid arthritis but not PsA in Japan.

The Japanese guidance for use of bDMARDs in PsO recommends using bDMARDs early in the PsA disease course to prevent joint destruction [28]. The first bDMARD was prescribed at a median duration of 1 to 2 months after a PsA diagnosis in the Japanese subgroup, which was earlier than in the overall LOOP population [14]. Moreover, the rate of TNFi prescription was comparable for both clinical specialties in contrast to that of anti–IL-12/23 or other bDMARDs, which were prescribed more frequently by dermatologists. Rheumatologists/orthopedists preferred the use of TNFi’s (adalimumab and infliximab), given that these were the first approved bDMARDs for the treatment of PsA in Japan in 2009 [29]. In contrast, dermatologists are more accustomed to administering IL-17 antagonists (secukinumab, ixekizumab, and brodalumab) and IL-12/23 antagonists (ustekinumab), which were approved for the treatment of PsO and PsA in Japan between 2011 and 2016 [30,31], likely resulting in higher prescription rates for anti–IL-12/23 and other bDMARDs by this specialty.

Disease activity was generally well controlled and comparable across rheumatology/orthopedic and dermatology settings in Japan. Among the indicators of disease activity, SJC 66 was estimated higher in the rheumatology/orthopedic *vs*. dermatology setting (2.6 *vs*. 1.4, p = 0.043), and the number of nails with psoriatic changes was higher in the dermatology *vs*. rheumatology/orthopedic setting (4.1 vs 7.9, p = 0.0038). Similarly, TJC 68 was numerically higher in the rheumatology/orthopedic *vs*. the dermatology setting, and BSA was higher in the dermatology *vs*. the rheumatology/orthopedic setting. This may reflect that patients with severe joint symptoms tend to visit rheumatologists, while patients with severe skin and nail symptoms tend to visit dermatologists. This is in contrast to the findings in the overall LOOP population in which the majority of disease activity assessments were significantly worse in the dermatology than in the rheumatology settings, likely due to the difference in overall treatment reflected in lower use of csDMARDs and TNFi [14]. The number of csDMARD prescriptions was higher for rheumatologists/orthopedists in the Japanese subgroup analysis compared with that in the overall LOOP analysis. It was also shown that, in the Japanese subgroup analysis, a higher proportion of bDMARD, especially anti–IL-12/23 and other bDMARDs, was prescribed by dermatologists compared with rheumatologists/orthopedists which may have resulted in adequate control of disease activity.

Previous studies demonstrated that a multidisciplinary approach to managing PsA did not only lead to improved rates of early diagnosis, improved treatment outcomes, and increased patient and physician satisfaction, but also improved management of difficult-to-diagnose and difficult-to-treat patients [11–13]. Moreover, the need for a referral could be reduced if a single collaborating unit managed patient with PsA, thereby shortening the time to diagnosis and initiation of optimal treatment [7]. To improve collaboration between dermatology and rheumatology units, a Swiss group developed specific recommendations in 2015 for the management of PsA on the basis of an interdisciplinary consensus following identification of important domains from a systematic literature search and 3 rounds of Delphi exercise [32]. Although the importance of PsA among patients with PsO, with respect to its impact on current disease activity, was recognized by this exercise, no consensus was reached on the gold standard for the treatment of PsA.

The Japanese PsA guidelines state that joint symptoms in patients with PsO should be evaluated by rheumatologists, because symptom of joint pain may stem from causes other than PsA [33]. Furthermore, dermatologists should play a significant role in the evaluation and treatment of skin and nail symptoms [33]. For PsA, treatment should be individualized based on a patient’s symptoms, while also considering comorbidities and complications. This study highlighted treatment modalities, including clinical history, for PsA in rheumatology/orthopedic and dermatology setting in daily clinical practice under the Japanese health care system. Mutual understanding between rheumatologists/orthopedists and dermatologists may help improve collaboration to optimize management of patients with PsA.

One of the limitations of the current subgroup analysis of Japanese treatment centers was that all except 2 participating institutions were university hospitals with an existing collaborative approach. Thus, the extent of existing collaboration outside of these institutions to manage patients with PsA remains unclear. In addition, all the dermatology sites were certified by the Japanese Dermatological Association (JDA) to use bDMARD. In Japan, JDA certification is required to prescribe bDMARDs to patients with PsO or PsA in a dermatology institution [30]. Therefore, dermatologists working in non–JDA-certified dermatology institutions can only prescribe csDMARDs or systemic steroids. The number of JDA-certified institutions is limited (525 sites in 2017) [34]; therefore, the study results do not reflect the general prescribing trend of dermatologists in Japan. Furthermore, data from patients who started receiving medical treatment prior to a confirmed diagnosis (mean time from PsA diagnosis to first csDMARD: rheumatology setting, −0.9 months; dermatology setting, −2.9 months) at sites participating in the current analysis may have impacted the time from the onset of inflammatory musculoskeletal symptoms to PsA diagnosis and the evaluation of disease severity at the time of confirmed diagnosis.

Overall, results from this subgroup analysis of Japanese patients from the LOOP study showed differences in treatment approach and patient characteristics between rheumatology/orthopedic and dermatology setting. These results lend further support to a tighter collaboration between rheumatologists/orthopedists and dermatologists to optimize and individualize the management of patients with PsA. Furthermore, an integrated dermo-rheumatologic approach could potentially aid in early diagnosis and timely management, thereby lowering disease activity and burden in patients with PsA.

## Supporting information

S1 Checklist TREND statement checklist. (PDF)

S1 File (PDF)
