# Supplementary material for: LOOP, a cross-sectional, observational study investigating the clinical specialty setting as a determinant of disease management in psoriatic arthritis: Subgroup analysis results from Japan
Source: PLoS One. 2021 Jan 27;16(1):e0245954. doi: 10.1371/journal.pone.0245954 (PMC7840027; doi:10.1371/journal.pone.0245954)
Supplement: S1 File — (PDF) [file pone.0245954.s002.pdf]

1. Title Page

AbbVie

Observational Research Plan Nr. 10 751

LOOP - Cross-sectionaL ObservatiOnal study evaluating clinical specialty setting as determinant of management of Patients with Psoriatic Arthritis

Product Name:	None
Type of Study:	Observational Research
Date:	06 Nov 2015
Biometrics:	GKM Gesellschaft für Therapieforschung mbH<br>Lessingstrasse 14<br>80336 München<br>Germany
Sponsor(s):	AbbVie Affiliates in the participating countries<br>Study co-ordinating country is AbbVie France

This study will be conducted in compliance with this research plan and all applicable regulatory requirements

Confidential Information
No use or disclosure outside AbbVie is permitted without prior written authorization from AbbVie

2. Table of Contents

1. TITLE PAGE 1

2. TABLE OF CONTENTS 2

3. INTRODUCTION 3

4. RATIONALE 4

5. STUDY OBJECTIVES 5

6. OBSERVATIONAL PLAN 5

6.1. STUDY CONDUCT 5

6.1.1. Patient Selection Criteria 7

6.1.2. Site Selection Criteria 7

6.1.3. Number of patients 7

6.2. STUDY DURATION 7

6.3. DESCRIPTION OF ACTIVITIES 7

6.4. EVENT REPORTING 10

7. ETHICS AND QUALITY 10

7.1. SOURCE DOCUMENTS 10

7.2. QUALITY ASSURANCE 11

8. DATA ANALYSIS PLAN 11

8.1. ENDPOINTS 11

8.2. PLAN FOR STATISTICAL ANALYSIS 11

8.3. SAMPLE SIZE CALCULATION 12

9. FINAL REPORT AND PUBLICATIONS 12

10. REFERENCES 13

11. RESEARCH PLAN SIGNATURE PAGE 16

12. AFFILIATE AGREEMENT PAGE 17

13. APPENDICES 19

APPENDIX A: SF12v2 19

APPENDIX B: HAQ-DI 22

APPENDIX C: WPAI:PSA 24

APPENDIX D: DLQI 25

Confidential Information
No use or disclosure outside AbbVie is permitted without prior written authorization from AbbVie

3. Introduction

Psoriatic arthritis (PsA) is a chronic systemic immune mediated inflammatory disease characterized by the association of arthritis and periarticular inflammation with skin psoriasis. PsA is regularly allied with plaque psoriasis and presents as asymmetric mono-/oligoarthritis or symmetric polyarthritis, distal interphalangeal (DIP) joint involvement and/or axial disease. Other common PsA features are enthesitis, dactylitis and psoriatic nail dystrophy (1-6).

PsA occurs in around 30% of patients with psoriasis, while its population prevalence estimates range from 0.1-0.5% worldwide (7, 8). Typically, psoriasis precedes the development of arthritic symptoms by several years (9, 10). However, the clinical patterns of PsA are heterogeneous and may vary in an individual patient over time, which renders recognition of the disease by non-rheumatologists and patients challenging (6, 11, 12).

The medical and economic burden of PsA could be significant for the patient and society. Up to 30% of PsA patients exhibit a progressive, disabling form of arthritis and almost 70% of PsA patients seen in clinics have evidence of erosive disease (2, 3, 11, 12). PsA may thus lead to impaired physical function, decreased quality of life, work disability, and is also associated with certain comorbidities.
Initially, the recognition of comorbidities has been limited to the classical features or extra-articular manifestations (EAMs) of spondyloarthritis, such as inflammatory bowel disease and uveitis (5, 11, 13-15). Over the past few years, it has been increasingly recognized that an increased prevalence of both novel and traditional cardiovascular risk factors as well as of established cardiovascular disease, such as ischaemic heart disease and cerebrovascular disease, represents a major source of morbidity and mortality in PsA (16-18). The burden of cardiovascular disease is larger in PsA than in psoriasis itself and may be reduced by systemic anti-inflammatory treatment (19, 20). Further, obesity, metabolic syndrome and type II diabetes mellitus, which occur frequently in PsA patients, may negatively affect disease activity and response to therapy (21-23). Of note, increased incidence rates of malignancies other than skin cancer, and similar to that in patients with rheumatoid arthritis, were found in the North American CORRONA database (24).

To improve long-term outcomes in patients with PsA, early and appropriate treatment seems to be important, starting with timely diagnosis. It has been shown that patients with more than a 6-month delay only in rheumatology consultation had more structural damage and worse physical function than those who visited a rheumatologist within 6 months of symptom onset (25). This observation is supported by two former studies: a delay in PsA diagnosis for more than 1 year was associated with worse physical function (26), and patients seen within the first 2 years of PsA had clinically less severe disease and less radiographic progression than those who presented themselves 2 years after symptom onset (27).
Effective treatment of PsA should generally consist of disease modifying antirheumatic drugs (DMARDs), first conventional systemic DMARDs (csDMARDs), followed by biologic DMARDs (bDMARDs) (28-30). There is however lack of evidence that csDMARDs inhibit radiographic progression in PsA. Also a direct proof that early versus delayed treatment with csDMARDs is beneficial in the long-term is missing in PsA (31). Concerning bDMARDs, it has been shown that patients who received an anti-tumor necrosis factor agent (anti-TNF) within 2 years of PsA duration experienced greater improvements in arthritis scores and patient reported outcomes (PROs) than those with more than 2 years of PsA (32). Further, the first randomized controlled trial in spondyloarthritis (TICOPA) that compared a treat to target approach with standard of care in early PsA patients demonstrated significantly improved joint and skin outcomes by the tight disease control approach.

Confidential Information
No use or disclosure outside AbbVie is permitted without prior written authorization from AbbVie

That consisted of targeting minimal disease activity (MDA) and stepwise treatment algorithm starting with methotrexate as the first cdDMARD and treatment escalation every 3 months if the target of MDA was not achieved. At the end of the TICOPA trial, 37% of patients were on a bDMARD in the tight control arm versus 7.6% only in the standard care arm (33).

A number of international and national recommendations for the management of PsA and corresponding treatment algorithms have been published in the past few years in both rheumatology and dermatology (28-31, 34, 35). Ideally, management of PsA should address joints and skin as well as extra-articular manifestations and comorbidities (34, 35). It has been demonstrated that PsA patients receiving a multidisciplinary care, involving both rheumatologists and dermatologists in a US clinic, were more likely to receive systemic medication (25% vs 15%) and be treated with a biologic agent (37% vs 16%) than in prior unidisciplinary care (36).

While consensus is evident among the recommendations on the main treatment principles, the extent to which they are followed by practicing physicians is unknown. In reality it appears that many patients with PsA are not diagnosed, or are undertreated or not treated systematically (34, 37). A recent large population based multinational survey conducted in North America and Europe revealed that approximately 15% of patients with PsA had not seen a health care provider in the past year, and almost 60% were not being treated for their joint disease (38).

4. Rationale

Evidence suggests that timely and effective management can improve long-term outcomes in patients with PsA. The factors influencing management decisions in PsA are not well understood. In the field of diabetes mellitus, the interactions of patient, physician and systemic factors were found to have implications for the implementation of a diabetes management model (39).

Early diagnosis of PsA is the first and crucial step in effective management of PsA, The detection of inflammatory musculoskeletal symptoms drives PsA diagnosis. The clinical patterns of PsA are heterogeneous and may vary in an individual patient over time, which renders recognition of the disease by non-rheumatologists and patients challenging, leading to a delay in diagnosis and worse long term outcomes.
Therefore, the time of musculoskeletal symptoms onset is selected in this study as the anchor date for describing different management steps in patients with PsA. In order to assess this aspect in the overall PsA population both patients with established and suspected PsA diagnosis will be asked to participate in the study. Medical care of PsA patients are provided by various clinical specialties, such as rheumatologists, dermatologists, other specialists and primary care practices. In order to describe the overall PsA population, which includes patients with mild disease that are not consulting physicians with rheumatology or dermatology expertise, the study aims to include PsA patients from all specialties providing medical care.

This study is expected to provide important insights into the factors influencing management of PsA, which may aid planning improvements in the standards of care in this chronic systemic disease.

Confidential Information
No use or disclosure outside AbbVie is permitted without prior written authorization from AbbVie

5. Study Objectives

Primary objective:

- To evaluate the association between the clinical specialty setting and time from inflammatory musculoskeletal symptom onset to PsA diagnose and to different management steps in patients with a confirmed PsA diagnosis

Secondary objectives:

- To explore the association between the timing of various management steps and current disease activity and burden in patients with a confirmed PsA diagnosis
- To describe the disease characteristics and comorbidities of patients whose suspected PsA diagnosis is not confirmed through a current routine rheumatological and dermatological assessment

This is an observational study. It is not designed to identify or quantify a safety hazard relating to an authorized medicinal product.

6. Observational Plan

6.1. Study Conduct

This study will be performed in a cross-sectional, observational, multi-country, multi-center format in approximately 15 countries across different geographical regions.

The study population will consist of patients who are 18 years or older, have been diagnosed with suspected or established Psoriatic Arthritis (PsA) and attending a routine clinical visit at the participating clinical sites. Suspected PsA diagnosis is based on clinical judgement of the recruiting physician.

In order to describe the overall PsA population, all specialties providing medical care of PsA patients will be included. The study will be conducted in a convenience sample recruited at rheumatology, dermatology, other specialist and primary care practices. These are providing medical care for 37-38% (rheumatology), 22-28% (dermatology), 7-12% (other specialists) and 28% (primary care practices) of PsA patients, respectively, depending on the country (38). Other specialties may include orthopedists, ophthalmologists, physiatrists and podiatrists according to the standard practice in the respective country (34). For the sake of convenience, other specialist and primary care practices will be referred to as non-rheum/non-derm sites in this research plan.

Patients with a suspected or an established diagnosis of PsA scheduled for routine visit at the recruiting site (dermatologist, rheumatologist or a non-rheum/non-derm site) can be offered the opportunity to participate in the study. Patients will be recruited on a consecutive basis. Patient characteristics, relevant co-morbidities and medical history, disease, diagnostic and treatment data will be documented in the Data Recording Form (DRFs) for data available in the medical charts.

Confidential Information
No use or disclosure outside AbbVie is permitted without prior written authorization from AbbVie

For the management of PsA patients a regular collaboration between different specialties is advised by established clinical recommendations (28-31, 34, 35). In order to ensure most accurate and standardized assessments of joint and skin scores as recommended in guidelines (29), the recruiting site will advise a consulting visit with a dermatologist and/or a rheumatologist for a routine PsA disease assessment. The consulting visit for a patient recruited at a rheumatology site will be with a dermatologist, for a patient recruited at a dermatology site, the consulting visit will be with a rheumatologist. Patients recruited at non-derm/non-rheum sites will be advised for consulting visits with a dermatologist and a rheumatologist, depending on country practice, based on the respective specialty assessments specified in DRF module 2 and module 3.

Consulting visits for current PsA disease assessments by dermatologist and/or rheumatologists usually happens between a 12 weeks period after a routine visit at the recruiting site and will be documented in the Data Recording Forms (see Figure 1). Treatment, procedures and diagnostic methods will follow physicians' routine clinical practice.

Validated patient questionnaires will be used to evaluate the current disease activity and burden.
No patient identifiable information will be captured.

To assess the generalizability of the data enrolled, the physician will be asked to document on a separate Data Reporting Form (DRFs) the number of patients who are eligible but refuse to participate in the study. Only age ranges (<30 years, 30-45 years, > 45 years) and reason(s) for not participating (no time, not interested, does not speak the language of the questionnaires provided) will be collected.

Figure 1: Study schematic

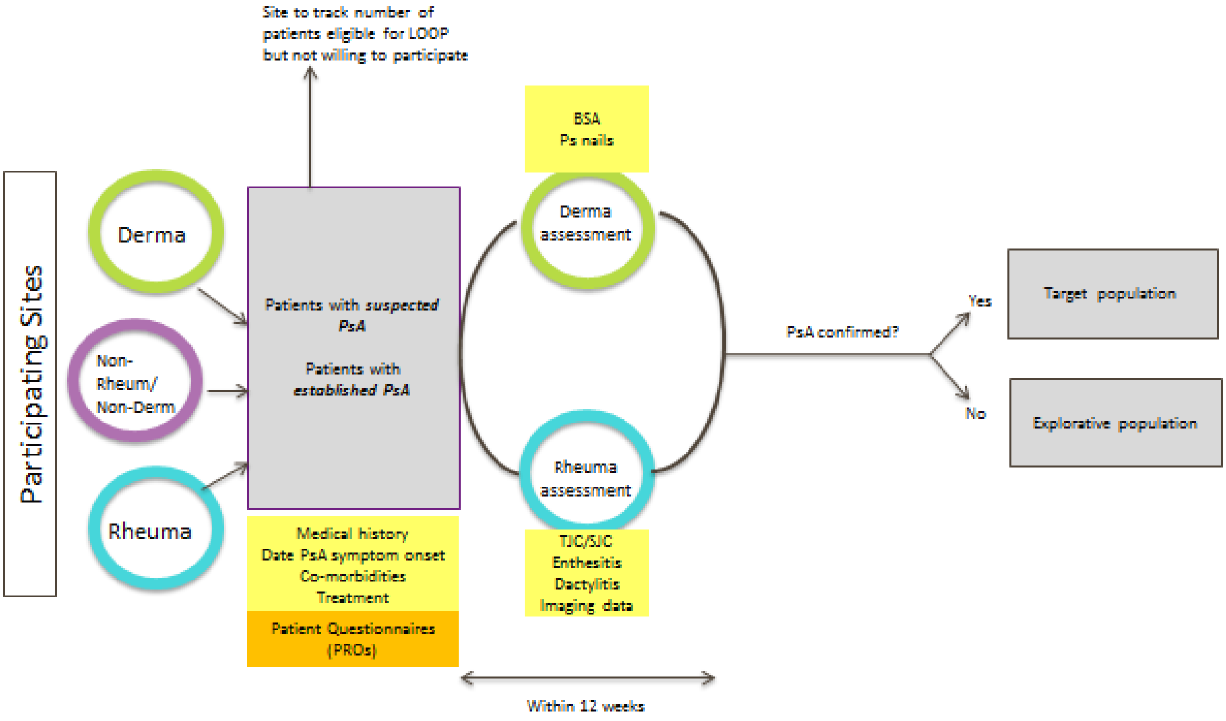

6.1.1. Patient Selection Criteria

Inclusion criteria
Patients attending a routine visit at a rheumatologist, dermatologist or non-rheum/non-derm site, who fulfill the following selection criteria, can be included:

- Diagnosed with suspected or established PsA
- Female or male
- Age of 18 years or older
- Signed a patient authorization form to use and disclose personal health information (or informed consent, where applicable)
- Able to read and understand patients questionnaires in provided language

No formal Exclusion criteria exist.

6.1.2. Site Selection Criteria

Rheumatology, dermatology and non-Rrheum/non-derm sites will be selected based on their patient population targeted for this study and their ability to appropriately conduct the study. The non-rheum/non-derm sites may include primary care practices, orthopedists, ophthalmologists, physiatrists and podiatrists according to the standard practice in the respective country.
The sample of sites will be recruited to be broadly representative of the settings providing medical care for PsA patients in the respective country.

6.1.3. Number of patients

Approximately 1.200 patients will be included in the study. The study will cover sites in around 15 countries in Western and Eastern Europe, Latin America and Asia.

6.2. Study Duration

This is a cross-sectional, observational study. The inclusion period will be approximately 16-18months.

6.3. Description of Activities

For the management of PsA patients a regular collaboration between different specialties is advised by established clinical recommendations (28-31, 34, 35). Consulting visits for current PsA disease assessments by dermatologist and/or rheumatologists, depending on the recruiting site, usually happens between a 12 weeks period after a routine visit at the recruiting site.

Confidential Information
No use or disclosure outside AbbVie is permitted without prior written authorization from AbbVie

The following will be documented:

Recruiting site – Module 1

- Socio-demographic information
- PsA symptoms and diagnosis
- Relevant medical history
- Relevant co-morbidities
- PsA treatment
- Administration of patient questionnaires

If the recruiting site is a rheumatologist, the rheumatology module 2 will be used in addition.
If the recruiting site is a dermatologist, the dermatology module 3 will be used in addition.

Rheumatology Module 2

- TJC/SJC (66/68)
- Enthesitis count
- Dactylitis count
- PtGA
- CRP/ESR (if available)
- Imaging data (if available)
- Components of ASDAS (for patients with back pain only)

Dermatology Module 3

- BSA
- PGA
- Psoriatic nails (count)
- Musculoskeletal signs

This study will assess PsA disease burden by using validated Patient Reported Outcome (PRO) instruments. Validated translations of these patient questionnaires will be administered as paper format.

The following questionnaires will be used:

- **SF12v2**: This Quality of Life survey asks for the patient's view about his/her health, how the patient feels and how well he/she is able to do the usual activities. The questionnaire consists of 12 questions, should require approximate 8 minutes to complete and is presented in Appendix A.
- **HAQ-DI** (Health Assessment Questionnaire Disability Index): It consists of 15 questions assessing the patients' physical function and health-related quality of life. The questionnaire should require approximate 12 minutes to complete and is presented in Appendix B.
- **WPAI PsA** (Work Productivity and Activity Impairment Questionnaire): It consists of 6 questions assessing the impact of the disease on the ability of the patient to work and perform normal daily activities. The questionnaire should require approximate 7 minutes to complete and is presented in Appendix C.
- **DLQI** (Dermatology Life Quality Index): the aim of this questionnaire is to measure how much the skin problem has affected the life of the patient. The questionnaire consists of 10 questions which should require approximate 5 minutes to complete and is presented in Appendix D.

Confidential Information
No use or disclosure outside AbbVie is permitted without prior written authorization from AbbVie

Table 1. Schedule of Assessments

Assessments	General Data	Musculoskeletal Signs	Skin Signs
	Recruiting site	Rheumatologist	Dermatologist
Patient authorization/Informed consent	X		
Socio-demographic data	X		
PsA symptoms	X		
Relevant medical history	X		
Relevant co-morbidities	X		
PsA treatment	X		
Confirmation of PsA (per country Health Care System)	X	X	X
TJC/SJC (66/68)		X	
Enthesitis count		X	
Dactylitis count		X	
PtGA		X	
CRP /ESR (if available)		X	
Imaging data (if available)		X	
Components of ASDAS (for patients with back pain only)		X	
BSA		X	X
PGA			X
Psoriatic nails (count)			X
Musculoskeletal signs			X
Confirmation of psoriasis			X

Patient Questionnaires (PROs)	
HAQ-DI	X
SF-12v2	X
DLQI	X
WPAI PsA	X

Confidential Information
No use or disclosure outside AbbVie is permitted without prior written authorization from AbbVie

6.4. Event Reporting

This is an observational research study not designed to identify or quantify a safety hazard relating to an AbbVie authorized product.

If a patient reports a product-related event (e.g. suspected adverse reaction or product complaint) to his/her healthcare professional during the data collection period or the healthcare professional identifies a product-related event, which is considered related to any AbbVie authorized product, the event should be reported to AbbVie. Any product-related events considered to be related to a non-AbbVie product should be reported in accordance with local laws and regulations to the relevant Regulatory Authority and/or drug marketing authorisation holder. In the event of a pregnancy occurrence in a patient during the data collection period, the pregnancy should be reported to AbbVie.

Patient Reported Outcome (PRO) or quality of life questionnaires data are not considered a potential source of adverse reactions. However, participating sites should review the PRO or questionnaire(s) data and if a possible product-related event (including a suspected adverse reaction) is noted, the HCP must determine whether the event is related to an AbbVie authorized product and if so, it should be reported to AbbVie

7. Ethics and Quality

This observational study will be run in compliance with local laws and regulations. Notification/ submission to the responsible Ethics Committee, Health Institutions and/or Competent Authorities will be done as required by local laws and regulations.

Written patient authorization to use and/or disclose his/her anonymized health data (and informed consent where applicable) will be obtained prior to patient inclusion.

To maintain subject confidentiality, no demographic data that can identify the patient will be collected (e.g. initials, date of birth), only the patient age will be collected. In order to protect patient's identity, a unique number will be assigned to each patient and related study records.

Data Recording Forms (DRF) in English and patient questionnaires in local language will be provided to each site in paper format coded with a unique patient number. The participating site will be asked to send the filled out Forms/Questionnaires regularly to the AbbVie Affiliate office in the respective country (see section 11 Affiliate Agreement page).

7.1. Source Documents

The investigator must maintain source documents for each patient in the study, consisting of medical records containing demographic data, medical, treatment and diagnostic documentation.

Confidential Information
No use or disclosure outside AbbVie is permitted without prior written authorization from AbbVie

7.2. Quality Assurance

The sites will be instructed in the research plan, the handling of the paper DRFs and the administration of the patient questionnaires.
Continuous oversight of the observational study will be performed by AbbVie or a CRO working on behalf of AbbVie.

8. Data Analysis Plan

8.1. Endpoints

Primary Endpoints

- Time from inflammatory musculoskeletal symptom onset to PsA diagnosis
- Time from PsA diagnosis to first csDMARD
- Time from PsA diagnosis to first bDMARD
- Time from first csDMARD to first bDMARD

Secondary Endpoints

- PsA disease activity
  - Joint disease activity (TJC/SJC, enthesitis count and dactylitis count, axial involvement by ASDAS
  - Skin disease activity (PGA, BSA, psoriatic nails count)
- Disease burden :
  - Physical function (HAQ-DI score)
  - Quality of life (SF12v2.0 score)
  - Work productivity (WPAI scores)
  - DLQI score

8.2. Plan for Statistical Analysis

Only patients with a confirmed diagnosis of PsA will be eligible for statistical analysis. The exact definition of the analysis populations will be detailed in a separate statistical analysis plan.
Collected data will be summarized by descriptive statistics. Quantitative variables will be reported as absolute N, mean, standard deviation, median, 1st and 3rd quartiles and minimum and maximum. Qualitative variables will be reported as absolute and relative frequency distributions.
Research outcomes of primary and secondary interest will be analyzed using descriptive statistics overall and in subgroups defined by explanatory variables (disease severity perception by patients and physicians and clinical specialty). Effects will be estimated depending on the scale of the endpoint by use of mean differences or ORs with respective 95% confidence interval. The effect estimates will be calculated from simple regression

models run separately for each of the explanatory variables and thereafter in multiple regression models with variable selection to identify and assess important explanatory variables. Depending on the scale of the dependent variable, linear or logistic models will be used.
PsA clinical manifestation and disease burden will be analyzed using descriptive statistics. Regression analyses will be done as described above, using time from symptom onset to PsA diagnosis, time from PsA diagnosis to first csDMARD, first bDMARD and from first csDMARD to first bDMAD as explanatory variables.

8.3. Sample Size Calculation

The factor that is most likely to influence the PsA management components of interest is clinical specialty. However, there is no scientific basis currently to judge the effect size.
So this will be an exploratory study and objectives are exploratory in nature (non-confirmatory) and no adjustment for multiplicity will be made.
With 400 evaluable patients in rheum and derm sites, the study has 90% power to detect a difference in means between the two specialties with a two-sample t-test at a significance level of 5% assuming an effect size of 0.229. With 400 evaluable patients in rheum or derm sites and 200 evaluable patients in non-rheum/non-derm sites, the study has 90% power to detect a difference in means between rheum vs. non-rheum/non-derm or derm vs. non-rheum/non-derm, respectively, with a two-sample t-test at a significance level of 5% assuming an effect size of 0.281.
The estimated drop-out rates due to non-confirmed PsA diagnosis are 10% in rheum, 15% in derm and 25% in non-rheum/non-derm sites. Hence, a total number of approximately 1200 patients (450:480:270 in rheum:derm:non-derm/non-rheum, respectively) has to be recruited in order to achieve 1000 evaluable patients (400:400:200 in rheum:derm:non-derm/non-rheum, respectively).

9. Final Report and Publications

At the end of this observational study, a report will be written by AbbVie. This report will contain a description of the objectives of the study, the methodology and its results and conclusions. The completed Data Recording Forms, patient questionnaires and the study report are the confidential property of AbbVie and may not be released to unauthorized people in any form (publications or presentations) without the express written approval from AbbVie.

Confidential Information
No use or disclosure outside AbbVie is permitted without prior written authorization from AbbVie

10. References

1. Moll JMH, Wright V. Psoriatic arthritis. Semin Arthritis Rheum 1973;3:55–78.
2. Gladman DD, Shuckett R, Russell ML, Thorne JC, Schachter RK. Psoriatic arthritis (PSA): an analysis of 220 patients. Q J Med 1987;62:127–41
3. Torre Alonso JC, Rodriguez Perez A, Arribas Castrillo JM, Ballina Garcia J, Riestra Noriega JL, Lopez Larrea C. Psoriatic arthritis (PA): a clinical, immunological and radiological study of 180 patients. Br J Rheumatol. 1991 Aug;30(4):245–250.
4. Helliwell PS, Taylor WJ. Classification and diagnostic criteria for psoriatic arthritis. Ann Rheum Dis. 2005;64(Suppl. II):ii, 3–8.
5. Gladman DD. Psoriatic arthritis. Dermatol Ther. 2009;22:40–55.
6. Coates LC, Helliwell PS. Classification and categorization of psoriatic arthritis. Clin Rheumatol 2008;27: 1211-6.
7. Gladman DD, et al. J Rheumatol 2009; 36: 4–8.
8. Mease PJ, Gladman DD, Papp KA et al. Prevalence of rheumatologist-diagnosed psoriatic arthritis in patients with psoriasis in European/North American dermatology clinics. J Am Acad Dermatol 2013; 69:729–35.
9. Gladman DD. Psoriatic arthritis. Rheum Dis Clin North Am1998;24:829–43.
10. Espinoza LR, van Solingen R, Cuellar ML, Angulo J. Insights into the pathogenesis of psoriasis and psoriatic arthritis. Am J Med Sci. 1998 Oct;316(4):271-6.
11. Kane D, Stafford L, Bresnihan B, FitzGerald O. A prospective, clinical and radiological study of early psoriatic arthritis: an early synovitis clinic experience. Rheumatol 2003;42:1460–8.
12. McHugh NJ, Balachrishnan C, Jones SM. Progression of peripheral joint disease in psoriatic arthritis: A 5-yr prospective study. Rheumatology (Oxford) 2003; 42: 778-783.
13. Rapp SR, Feldman SR, Exum ML, et al. Psoriasis causes as much disability as other major medical diseases. J Am Acad Dermatol. 1999;41:401–7
14. Scarpa R, Manguso F, D'Arienzo A, D'Armiento FP, Astarita C, Mazzacca G, Ayala F. Microscopic inflammatory changes in colon of patients with both active psoriasis and psoriatic arthritis without bowel symptoms. J Rheumatol. 2000;27:1241–1246.
15. Haroon M, Gallagher P, Heffernan E, FitzGerald O. High prevalence of metabolic syndrome and of insulin resistance in psoriatic arthritis is associated with the severity of underlying disease. J Rheumatol. 2014;41(7):1357-65.
16. Jamnitski A et al. Ann Rheum Dis 2013; 72: 211-6. Jamnitski A et al, CV comorbidities in patients with psoriatic arthritis, AnnRheumDis, 2013, 72: 211-6
17. Husted JA, Thavaneswaran A, Chandran V, et al. Cardiovascular and other comorbidities in patients with psoriatic arthritis: a comparison with patients with psoriasis. Arthritis Care Res (Hoboken) 2011;63:1729–35
18. Armstrong AW, Harskamp CT, Armstrong EJ. The association between psoriasis and hypertension: a systematic review and meta-analysis of observational studies. 2013;31(3):433-43.
19. Edson-Heredia E, Zhu B, Lefevre C, Wang M, Barrett A, Bushe CJ, Cox A, Wu JJ, Maeda-Chubachi T. Prevalence and incidence rates of cardiovascular, autoimmune, and other diseases in patients with psoriatic or psoriatic arthritis: a retrospective study using Clinical Practice Research Datalink. J Eur Acad Dermatol Venereol. 2015 May;29(5):955-63. Epub 2014 Oct 28.

Confidential Information
No use or disclosure outside AbbVie is permitted without prior written authorization from AbbVie

20. Ogdie A, Yu Y, Haynes K, Love TJ, Maliha S, Jiang Y, Troxel AB, Hennessy S, Kimmel SE, Margolis DJ, Choi H, Mehta NN, Gelfand JM. Risk of major cardiovascular events in patients with psoriatic arthritis, psoriasis and rheumatoid arthritis: a population-based cohort study. Ann Rheum Dis. 2015 Feb;74(2):326-32. Epub 2014 Oct 28.

21. Sharma A, Gopalakrishnan D, Kumar R, Vijayvergiya R, Dogra S. Metabolic syndrome in psoriatic arthritis patients: a cross-sectional study. Int J Rheum Dis 2013; 16: 667-73.

22. Dreiher J, Freud T, Cohen AD. Psoriatic arthritis and diabetes: A population-based cross-sectional study. Dermatol Res Pract 2013;2013:580404.

23. Coto-Segura P, Eiris-Salvado N, González-Lara L, Queiro-Silva R, Martinez-Camblor P, Maldonado-Seral C, García-García B, Palacios-García L, Gomez-Bernal S, Santos-Juanes J, Coto E.Br J Dermatol. 2013 Oct;169(4):783-93. doi: 10.1111/bjd.12473. Review.

24. Gross RL, Schwartzman-Morris JS, Krathen M, Reed G, Chang H, Saunders KC, Fisher MC, Greenberg JD, Putterman C, Mease PJ, Gottlieb AB, Kremer JM, Broder A. Arthritis Rheumatol. 2014 Jun;66(6):1472-81.

25. Haroon M et al. Ann Rheum Dis 2014 Feb 27. (Epub ahead of print).

26. Tillett W, Jadon D, Shaddick G, et al. Smoking and delay to diagnosis are associated with poorer functional outcome in psoriatic arthritis. Ann Rheum Dis 2013;72(8):1358-61

27. Gladman DD, Thavaneswaran A, Chandran V, Cook RJ (2011). Do patients with psoriatic arthritis who present early fare better than those presenting later in the disease ? Ann Rheum Dis; 70: 2152-4

28. L Gossec, J S Smolen, C Gaujoux-Viala, Z Ash, H Marzo-Ortega, D van der Heijde, O FitzGerald, D Aletaha, P Balint, D Boumpas, J Braun, F C Breedveld, G Burmester, J D Cañete, M de Wit, H Dagfinrud, K de Vlam, M Dougados, P Helliwell, A Kavanaugh, T K Kvien, R Landewé, T Luger, M Maccarone, D McGonagle, N McHugh, I B McInnes, C Ritchlin, J Sieper, P P Tak, G Valesini, J Vencovsky, K L Winthrop, A Zink, P Emery
European League Against Rheumatism recommendations for the management of psoriatic arthritis with pharmacological therapies Ann Rheum Dis 2012;71:4-12

29. Ritchlin CT, Kavanaugh A, Gladman DD, Mease PJ, Helliwell P, Boehncke WH, de Vlam K, Fiorentino D, Fitzgerald O, Gottlieb AB, McHugh NJ, Nash P, Qureshi AA, Soriano ER, Taylor WJ; Group for Research and Assessment of Psoriasis and Psoriatic Arthritis (GRAPPA). Treatment recommendations for psoriatic arthritis. Ann Rheum Dis. 2009 Sep;68(9):1387-94

30. Menter A, Korman NJ, Elmets CA, Feldman SR, Gelfand JM, Gordon KB, Gottlieb A, Koo JY, Lebwohl M, Leonardi CL, Lim HW, Van Voorhees AS, Beutner KR, Ryan C, Bhushan R.Guidelines of care for the management of psoriasis and psoriatic arthritis Section 6. Guidelines of care for the treatment of psoriasis and psoriatic arthritis: Case-based presentations and evidence-based conclusions. J Am Acad Dermatol. 2011 Jul;65(1):137-74

31. Coates LC, Kavanaugh A, Ritchlin CT; GRAPPA Treatment Guideline Committee. Systematic review of treatments for psoriatic arthritis: 2014 update for the GRAPPA. J Rheumatol. 2014 Nov;41(11):2273-6.

32. Kirkham B, de Vlam K, Li W, Boggs R, Mallbris L, Nab HW, Tarallo M. Early treatment of psoriatic arthritis is associated with improved patient-reported outcomes: findings from the etanercept PRESTA trial. Clin Exp Rheumatol. 2015 Jan-Feb;33(1):11-9. Epub 2014 Dec 22.

33. Coates LC, Moverley AR, McParland L, Brown S, Collier H, Law J, et al. Results of a randomised controlled trial comparing tight control of early psoriatic arthritis (TICOPA) with standard care: tight control improves outcome [abstract] Arthritis Rheum. 2013;65(Suppl):S346.

34. Helliwell P, Coates L, Chandran V, Gladman D, de Wit M, FitzGerald O, Kavanaugh A, Strand V, Mease PJ, Boehncke WH, Langley RG, Lubrano E, Maccarone M, Schulze-Koops H, Miceli-Richard C, Queiro R. Qualifying unmet needs and improving standards of care in psoriatic arthritis. Arthritis Care Res (Hoboken). 2014 Dec; 66(12):1759-66.

Confidential Information
No use or disclosure outside AbbVie is permitted without prior written authorization from AbbVie

35. Ogdie A, Schwartzman S, Eder L, Maharaj AB, Zisman D, Raychaudhuri SP, et al. Comprehensive treatment of psoriatic arthritis: Managing comorbidities and extra-articular manifestations. J Rheumatol 2014;41:2315-22.
36. Velez NF, Wei-Passanese EX, Husni ME, Mody EA, Qureshi AA. Management of psoriasis and psoriatic arthritis in a combined dermatology and rheumatology clinic. Arch Dermatol Res. 2012 Jan;304(1):7-13.
37. Tintle SJ, Gottlieb AB. Psoriatic arthritis for the dermatologist. Dermatol Clin, 2015 Jan; 33(1):127-48.
38. Lebwohl MG, Bachelez H, Barker J, Girolomoni G, Kavanaugh A, Langley RG, Paul CF, Puig L, Reich K, van de Kerkhof PC. Patient perspectives in the management of psoriasis: results from the population-based Multinational Assessment of Psoriasis and Psoriatic Arthritis Survey. J Am Acad Dermatol. 2014 May;70(5):871-81.e1-30
39. Brown JB, Harris SB, Webster-Bogaert S, Wetmore S, Faulds C, Stewart M. The role of patient, physician and systemic factors in the management of type 2 diabetes mellitus. Fam Pract. 2002 Aug;19(4):344-9.
40. McNett M, Goldenberg D, Schaefer C, Hufstader M, Baik R, Chandran A, Zlateva G. Treatment patterns among physician specialties in the management of fibromyalgia: results of a cross-sectional study in the United States. Curr Med Res Opin. 2011 Mar;27(3):673-83.
41. Ritchlin CT, Kavanaugh A, Gladman DD, Mease PJ, Helliwell P, Boehncke WH, de Vlam K, Fiorentino D, Fitzgerald O, Gottlieb AB, McHugh NJ, Nash P, Qureshi AA, Soriano ER, Taylor WJ; Group for Research and Assessment of Psoriasis and Psoriatic Arthritis (GRAPPA). Treatment recommendations for psoriatic arthritis. Ann Rheum Dis. 2009 Sep;68(9):1387-94.
42. Menter A, Korman NJ, Elmets CA, Feldman SR, Gelfand JM, Gordon KB, Gottlieb A, Koo JY, Lebwohl M, Leonardi CL, Lim HW, Van Voorhees AS, Beutner KR, Ryan C, Bhushan R.Guidelines of care for the management of psoriasis and psoriatic arthritis Section 6. Guidelines of care for the treatment of psoriasis and psoriatic arthritis: Case-based presentations and evidence-based conclusions. J Am Acad Dermatol. 2011 Jul;65(1):137-74
43. Richard MA, Barnetche T, Rouzaud M, Sevrain M, Villani AP, Aractingi S, Aubin F, Beylot-Barry M, Joly P, Jullien D, Le Maître M, Misery L, Ortonne JP, Cantagrel A, Paul C. Evidence-based recommendations on the role of dermatologists in the diagnosis and management of psoriatic arthritis: systematic review and expert opinion. J Eur Acad Dermatol Venereol. 2014;28 Suppl 5:3-12.
44. Taylor WJ et al. Arthritis Rheum 2006; 54:266515-73

Confidential Information
No use or disclosure outside AbbVie is permitted without prior written authorization from AbbVie

11. Research Plan Signature Page

AbbVie

Observational Research Plan Nr. 10 751

LOOP - Cross-sectionaL ObservatiOnal study evaluating clinical specialty setting as determinant of management of Patients with Psoriatic Arthritis

Approved by:

Fabiana Ganz — 17 November 2015

Fabiana Ganz
Associate Director Rheumatology
Global Medical Affairs
Scientific Study Lead

Date

17 NOV 2015

Peggy Schmitt
Clinical Project Manager
Abbvie France
Clinical & Operational Study Lead

Date

18. Nov. 2015

Bärbel Gross
Director Affiliate Research
Global Medical Affairs
Study Governance

Date

Confidential Information
No use or disclosure outside AbbVie is permitted without prior written authorization from AbbVie

12. Affiliate Agreement Page

AbbVie

Observational Research Plan Nr. 10 751

LOOP - Cross-sectionaL ObservatiOnal study evaluating clinical specialty setting as determinant of management of Patients with Psoriatic Arthritis

Sponsor:
Name Medical Director:
Address:
Address:
Country:
Phone:
Fax:

Local shipment address for DRFs/Questionnaires:
Name:
Address:
Address:
Country:
Phone:
Fax:

Confidential Information
No use or disclosure outside AbbVie is permitted without prior written authorization from AbbVie

Requirements for non-interventional studies per local laws and regulations:

Competent Authority approval ☐
Competent Authority notification ☐
Competent Authority involvement not required ☐

Ethics Committee approval ☐
Ethics Committee notification ☐
Ethics Committee involvement not required ☐

Written Patient Informed Consent required: ☐ No ☐ Yes

Regulatory requirements, other (if applicable):

____

Add name

____

Affiliate Medical Director, abbvie (add country)

____ _______________

Signature Date

Confidential Information
No use or disclosure outside AbbVie is permitted without prior written authorization from AbbVie

13. Appendices

Appendix A: SF12v2

Your Health and Well-Being

This survey asks for your views about your health. This information will help keep track of how you feel and how well you are able to do your usual activities. *Thank you for completing this survey!*

For each of the following questions, please mark an ☒ in the one box that best describes your answer.

1. In general, would you say your health is:

Excellent	Very good	Good	Fair	Poor
☐ 1	☐ 2	☐ 3	☐ 4	☐ 5

2. The following questions are about activities you might do during a typical day. Does <u>your health now limit you</u> in these activities? If so, how much?

	Yes, limited a lot	Yes, limited a little	No, not limited at all
a Moderate activities, such as moving a table, pushing a vacuum cleaner, bowling, or playing golf	☐ 1	☐ 2	☐ 3
b Climbing several flights of stairs	☐ 1	☐ 2	☐ 3

SAMPLE

SF-12v2® Health Survey © 1994, 2002 Medical Outcomes Trust and QualityMetric Incorporated. All rights reserved.
SF-12® is a registered trademark of Medical Outcomes Trust.
(SF-12v2® Health Survey Standard, United States (English))

Confidential Information
No use or disclosure outside AbbVie is permitted without prior written authorization from AbbVie

3. During the past 4 weeks, how much of the time have you had any of the following problems with your work or other regular daily activities as a result of your physical health?

	All of the time	Most of the time	Some of the time	A little of the time	None of the time
a Accomplished less than you would like	☐ 1	☐ 2	☐ 3	☐ 4	☐ 5
b Were limited in the kind of work or other activities	☐ 1	☐ 2	☐ 3	☐ 4	☐ 5

4. During the past 4 weeks, how much of the time have you had any of the following problems with your work or other regular daily activities as a result of any emotional problems (such as feeling depressed or anxious)?

	All of the time	Most of the time	Some of the time	A little of the time	None of the time
a Accomplished less than you would like	☐ 1	☐ 2	☐ 3	☐ 4	☐ 5
b Did work or other activities less carefully than usual	☐ 1	☐ 2	☐ 3	☐ 4	☐ 5

5. During the past 4 weeks, how much did pain interfere with your normal work (including both work outside the home and housework)?

Not at all	A little bit	Moderately	Quite a bit	Extremely
☐ 1	☐ 2	☐ 3	☐ 4	☐ 5

SAMPLE

SF-12v2® Health Survey © 1994, 2002 Medical Outcomes Trust and QualityMetric Incorporated. All rights reserved.
SF-12® is a registered trademark of Medical Outcomes Trust.
(SF-12v2® Health Survey Standard, United States (English))

Confidential Information
No use or disclosure outside AbbVie is permitted without prior written authorization from AbbVie

6. These questions are about how you feel and how things have been with you during the past 4 weeks. For each question, please give the one answer that comes closest to the way you have been feeling. How much of the time during the past 4 weeks…

	All of the time	Most of the time	Some of the time	A little of the time	None of the time
a Have you felt calm and peaceful?	☐ 1	☐ 2	☐ 3	☐ 4	☐ 5
b Did you have a lot of energy?	☐ 1	☐ 2	☐ 3	☐ 4	☐ 5
c Have you felt downhearted and depressed?	☐ 1	☐ 2	☐ 3	☐ 4	☐ 5

7. During the past 4 weeks, how much of the time has your physical health or emotional problems interfered with your social activities (like visiting with friends, relatives, etc.)?

All of the time	Most of the time	Some of the time	A little of the time	None of the time
☐ 1	☐ 2	☐ 3	☐ 4	☐ 5

Thank you for completing these questions!

SAMPLE

SF-12v2® Health Survey © 1994, 2002 Medical Outcomes Trust and QualityMetric Incorporated. All rights reserved.
SF-12® is a registered trademark of Medical Outcomes Trust.
(SF-12v2® Health Survey Standard, United States (English))

Confidential Information
No use or disclosure outside AbbVie is permitted without prior written authorization from AbbVie

Appendix B: HAQ-DI

HEALTH ASSESSMENT QUESTIONNAIRE (HAQ-DI)©

Name: ______________________________ Date: ______________

Please place an "x" in the box which best describes your abilities OVER THE PAST WEEK:

	WITHOUT ANY DIFFICULTY	WITH SOME DIFFICULTY	WITH MUCH DIFFICULTY	UNABLE TO DO
**DRESSING & GROOMING**				
Are you able to:				
Dress yourself, including shoelaces and buttons?	☐	☐	☐	☐
Shampoo your hair?	☐	☐	☐	☐
**ARISING**				
Are you able to:				
Stand up from a straight chair?	☐	☐	☐	☐
Get in and out of bed?	☐	☐	☐	☐
**EATING**				
Are you able to:				
Cut your own meat?	☐	☐	☐	☐
Lift a full cup or glass to your mouth?	☐	☐	☐	☐
Open a new milk carton?	☐	☐	☐	☐
**WALKING**				
Are you able to:				
Walk outdoors on flat ground?	☐	☐	☐	☐
Climb up five steps?	☐	☐	☐	☐

Please check any AIDS OR DEVICES that you usually use for any of the above activities:

☐ Devices used for Dressing (button hook, zipper pull, etc.)
☐ Built up or special utensils
☐ Crutches
☐ Cane
☐ Wheelchair
☐ Special or built up chair
☐ Walker

Please check any categories for which you usually need HELP FROM ANOTHER PERSON:

☐ Dressing and grooming ☐ Arising ☐ Eating ☐ Walking

- 1 -

Confidential Information
No use or disclosure outside AbbVie is permitted without prior written authorization from AbbVie

Please place an "x" in the box which best describes your abilities OVER THE PAST WEEK:

	WITHOUT ANY DIFFICULTY	WITH SOME DIFFICULTY	WITH MUCH DIFFICULTY	UNABLE TO DO
**HYGIENE**				
**Are you able to:**				
Wash and dry your body?	☐	☐	☐	☐
Take a tub bath?	☐	☐	☐	☐
Get on and off the toilet?	☐	☐	☐	☐
**REACH**				
**Are you able to:**				
Reach and get down a 5 pound object (such as a bag of sugar) from above your head?	☐	☐	☐	☐
Bend down to pick up clothing from the floor?	☐	☐	☐	☐
**GRIP**				
**Are you able to:**				
Open car doors?	☐	☐	☐	☐
Open previously opened jars?	☐	☐	☐	☐
Turn faucets on and off?	☐	☐	☐	☐
**ACTIVITIES**				
**Are you able to:**				
Run errands and shop?	☐	☐	☐	☐
Get in and out of a car?	☐	☐	☐	☐
Do chores such as vacuuming or yard work?	☐	☐	☐	☐

Please check any AIDS OR DEVICES that you usually use for any of the above activities:

☐ Raised toilet seat ☐ Bathtub bar ☐ Long-handled appliances for reach

☐ Bathtub seat ☐ Long-handled appliances in bathroom ☐ Jar opener (for jars previously opened)

Please check any categories for which you usually need HELP FROM ANOTHER PERSON:

☐ Hygiene ☐ Reach ☐ Gripping and opening things ☐ Errands and chores

- 2 -

Confidential Information
No use or disclosure outside AbbVie is permitted without prior written authorization from AbbVie

Your ACTIVITIES: To what extent are you able to carry out your everyday physical activities such as walking, climbing stairs, carrying groceries, or moving a chair?

COMPLETELY	MOSTLY	MODERATELY	A LITTLE	NOT AT ALL
☐	☐	☐	☐	☐

Your PAIN: How much pain have you had IN THE PAST WEEK?
On a scale of 0 to 100 (where zero represents "no pain" and 100 represents "severe pain"), please record the number below.

☐☐☐

Your HEALTH: Please rate how well you are doing on a scale of 0 to 100 (0 represents "very well" and 100 represents "very poor" health), please record the number below.

☐☐☐

- 3 -

Confidential Information
No use or disclosure outside AbbVie is permitted without prior written authorization from AbbVie

Appendix C: WPAI:PsA

Work Productivity and Activity Impairment Questionnaire: Psoriatic Arthritis V2.0 (WPAI:PsA)

The following questions ask about the effect of your Psoriatic Arthritis on your ability to work and perform normal daily activities. *Please fill in the blanks or circle a number, as indicated.*

1. Are you currently employed (working for pay)? _____ NO ___ YES
   If 'NO', tick "NO" and skip to question 6.

The next questions refer to the **past seven days**, not including today.

2. During the past seven days, how many hours did you miss from work because of problems associated with your Psoriatic Arthritis? *Include hours you missed on sick days, times you went in late, left early, etc., because of your Psoriatic Arthritis. Do not include time you missed to participate in this study.*

   _____ HOURS

3. During the past seven days, how many hours did you miss from work because of any other reason, such as annual leave, holidays, time off to participate in this study?

   _____HOURS

4. During the past seven days, how many hours did you actually work?

   _____HOURS *(If "0", skip to question 6.)*

WPAI:PsA V2.0 (UK English) 1

English for UK

5. During the past seven days, how much did your Psoriatic Arthritis affect your productivity while you were working?

Think about days you were limited in the amount or kind of work you could do, days you accomplished less than you would like, or days you could not do your work as carefully as usual. If Psoriatic Arthritis affected your work only a little, choose a low number. Choose a high number if Psoriatic Arthritis affected your work a great deal.

Consider only how much Psoriatic Arthritis affected productivity while you were working.

Psoriatic Arthritis had no effect on my work	0	1	2	3	4	5	6	7	8	9	10	Psoriatic Arthritis completely prevented me from working

CIRCLE A NUMBER

6. During the past seven days, how much did your Psoriatic Arthritis affect your ability to perform your normal daily activities, excluding your job?

By normal activities, we mean the usual activities you perform, such as working around the house, shopping, childcare, exercising, studying, etc. Think about times you were limited in the amount or kind of activities you could perform and times you accomplished less than you would like. If Psoriatic Arthritis affected your activities only a little, choose a low number. Choose a high number if Psoriatic Arthritis affected your activities a great deal.

Consider only how much Psoriatic Arthritis affected your ability to perform your normal daily activities, excluding your job.

Psoriatic Arthritis had no effect on my daily activities	0	1	2	3	4	5	6	7	8	9	10	Psoriatic Arthritis completely prevented me from performing my daily activities

CIRCLE A NUMBER

WPAI:PsA V2.0 (UK English) 2

English for UK

Confidential Information
No use or disclosure outside AbbVie is permitted without prior written authorization from AbbVie

Appendix D: DLQI

DERMATOLOGY LIFE QUALITY INDEX

The aim of this questionnaire is to measure how much your skin problem has affected your life OVER THE LAST WEEK. Please tick ☑ one box for each question.

1.	Over the last week, how itchy, sore, painful or stinging has your skin been?	Very much<br>A lot<br>A little<br>Not at all	
2.	Over the last week, how embarrassed or self conscious have you been because of your skin?	Very much<br>A lot<br>A little<br>Not at all	
3.	Over the last week, how much has your skin interfered with you going shopping or looking after your home or garden?	Very much<br>A lot<br>A little<br>Not at all	Not relevant
4.	Over the last week, how much has your skin influenced the clothes you wear?	Very much<br>A lot<br>A little<br>Not at all	Not relevant
5.	Over the last week, how much has your skin affected any social or leisure activities?	Very much<br>A lot<br>A little<br>Not at all	Not relevant
6.	Over the last week, how much has your skin made it difficult for you to do any sport?	Very much<br>A lot<br>A little<br>Not at all	Not relevant
7.	Over the last week, has your skin prevented you from working or studying?	Yes<br>No	Not relevant
	If "No", over the last week how much has your skin been a problem at work or studying?	A lot<br>A little<br>Not at all	
8.	Over the last week, how much has your skin created problems with your partner or any of your close friends or relatives?	Very much<br>A lot<br>A little<br>Not at all	Not relevant
9.	Over the last week, how much has your skin caused any sexual difficulties?	Very much<br>A lot<br>A little<br>Not at all	Not relevant
10.	Over the last week, how much of a problem has the treatment for your skin been, for example by making your home messy, or by taking up time?	Very much<br>A lot<br>A little<br>Not at all	Not relevant

Please check you have answered EVERY question. Thank you.

©AY Finlay, GK Khan, April 1992 www.dermatology.org.uk, this must not be copied without the permission of the authors.

Confidential Information
No use or disclosure outside AbbVie is permitted without prior written authorization from AbbVie
